# Homœopathic Medical College of Missouri

**Published:** 1888-04

**Authors:** 


HOMŒOPATHIC MEDICAL COLLEGE OF MISSOURI held its twenty-ninth annual commencement March 15th. An address was given by the Rev. John Snyder, D. D.

